# Advances and Applications in the Quest for Orthologs

**DOI:** 10.1093/molbev/msz150

**Published:** 2019-06-27

**Authors:** Natasha Glover, Christophe Dessimoz, Ingo Ebersberger, Sofia K Forslund, Toni Gabaldón, Jaime Huerta-Cepas, Maria-Jesus Martin, Matthieu Muffato, Mateus Patricio, Cécile Pereira, Alan Sousa da Silva, Yan Wang, Erik Sonnhammer, Paul D Thomas

**Affiliations:** 1 Department of Computational Biology, University of Lausanne, Lausanne, Switzerland; 2 SIB Swiss Institute of Bioinformatics, Lausanne, Switzerland; 3 Center for Integrative Genomics, University of Lausanne, Lausanne, Switzerland; 4 Department of Genetics, Evolution & Environment, University College London, London, United Kingdom; 5 Department of Computer Science, University College London, London, United Kingdom; 6 Applied Bioinformatics Group, Institute of Cell Biology and Neuroscience, Goethe University Frankfurt, Frankfurt, Germany; 7 Senckenberg Biodiversity and Climate Research Centre (BIK-F), Frankfurt, Germany; 8 LOEWE Centre for Translational Biodiversity Genomics (LOEWE-TBG), Frankfurt, Germany; 9 Experimental and Clinical Research Center, A Cooperation of Charité-Universitätsmedizin Berlin and Max Delbruck Center for Molecular Medicine, Berlin, Germany; 10 Max Delbruck Center for Molecular Medicine in the Helmholtz Association, Berlin, Germany; 11 Charité-Universitätsmedizin Berlin, Corporate Member of Freie Universität Berlin, Humboldt-Universität u Berlin, Berlin, Germany; 12 Berlin Institute of Health (BIH), Berlin, Germany; 13 Structural and Computational Biology Unit, European Molecular Biology Laboratory, Heidelberg, Germany; 14 Centre for Genomic Regulation (CRG), The Barcelona Institute of Science and Technology, Barcelona, Spain; 15 Universitat Pompeu Fabra (UPF), Barcelona, Spain; 16 ICREA, Barcelona, Spain; 17 Centro de Biotecnología y Genómica de Plantas, Instituto Nacional de Investigación y Tecnología Agraria y Alimentaria (INIA), Universidad Politécnica de Madrid (UPM), Madrid, Spain; 18 European Molecular Biology Laboratory, European Bioinformatics Institute, Wellcome Genome Campus, Hinxton, Cambridge, United Kingdom; 19 Eura Nova, Marseille, France; 20 Department of Microbiology and Cell Science, Institute for Food and Agricultural Sciences, University of Florida, Gainesville, FL; 21 Department of Microbiology and Plant Pathology, Institute for Integrative Genome Biology, University of California-Riverside, Riverside, CA; 22 Science for Life Laboratory, Department of Biochemistry and Biophysics, Stockholm University, Solna, Sweden; 23 Division of Bioinformatics, Department of Preventive Medicine, University of Southern California, Los Angeles, CA

**Keywords:** orthology, gene family, genome evolution

## Abstract

Gene families evolve by the processes of speciation (creating orthologs), gene duplication (paralogs), and horizontal gene transfer (xenologs), in addition to sequence divergence and gene loss. Orthologs in particular play an essential role in comparative genomics and phylogenomic analyses. With the continued sequencing of organisms across the tree of life, the data are available to reconstruct the unique evolutionary histories of tens of thousands of gene families. Accurate reconstruction of these histories, however, is a challenging computational problem, and the focus of the Quest for Orthologs Consortium. We review the recent advances and outstanding challenges in this field, as revealed at a symposium and meeting held at the University of Southern California in 2017. Key advances have been made both at the level of orthology algorithm development and with respect to coordination across the community of algorithm developers and orthology end-users. Applications spanned a broad range, including gene function prediction, phylostratigraphy, genome evolution, and phylogenomics. The meetings highlighted the increasing use of meta-analyses integrating results from multiple different algorithms, and discussed ongoing challenges in orthology inference as well as the next steps toward improvement and integration of orthology resources.

## Introduction

Orthologs are genes in different species that can be traced to the same gene in their last common ancestral genome (Fitch 1970). Fitch distinguished them from paralogs (genes in the same or different species, which arise from gene duplication events). This distinction is important because of the role of gene duplication in evolution of novel gene functions (Hurles 2004). The inference of orthologs is a cornerstone for comparative genomics, phylogenetics, and for the prediction of function in newly annotated genomes. Indeed, with the abounding number of genomes sequenced in recent decades, orthology relationships can now be inferred computationally.

However, developing accurate computational methods to infer orthologs is a challenging research problem: it requires reconstructing the gene content of ancestral genomes, and inference of how each gene family was shaped by its history of speciation, gene duplication, horizontal gene transfer (HGT), and gene loss. Thus, there is a need to improve orthology inference algorithms to deal with gene families with complex histories of gene duplications and loss, HGT, or domain gain or loss (Forslund et al. 2018). Additionally, computational tools used for orthology inference face challenges when dealing with an increasing number of genomes (Sonnhammer et al. 2014). Orthology is harder to detect at large evolutionary distances. Finally, methods for fair and accurate assessment of different inference methods (benchmarking) are required (Gabaldón et al. 2009).

Identifying and addressing these and other outstanding issues in orthology inference is a top priority of the Quest for Orthologs Consortium, which holds meetings biennially with researchers from around the world. As a result of these meetings and subsequent work from Consortium members, reference proteomes (the complement of all protein-coding genes in a genome) have been curated for a phylogenetically diverse set of genomes (Dessimoz et al. 2012), a consensus species tree has been developed through a review of recent literature (Boeckmann et al. 2015), and a benchmarking server has been implemented and assessed (Altenhoff et al. 2016). The Fifth Quest for Orthologs Meeting was paired with the SMBE Symposium on the Evolution of Gene Families (QFO5/SMBE-EGF) and held at the University of Southern California in June 2017 (https://sites.google.com/usc.edu/smbe-egf-2017; Last accessed April 2019). Together, these meetings featured over 30 speakers from ten different countries, addressing issues in orthology inference and its applications in evolutionary, biomedical, and agricultural sciences. Here, we review the highlights of the meetings concerning recent advances and challenges in the field of orthology inference, including its many applications. One of the main advances is that a large number of different orthology methods will be benchmarked on a regular basis, using a shared set of protein-coding genes. This will facilitate the comparison of different methods, as well as the use of multiple methods for identifying high-confidence consensus orthologs.

## Applications

**Fig 1:**
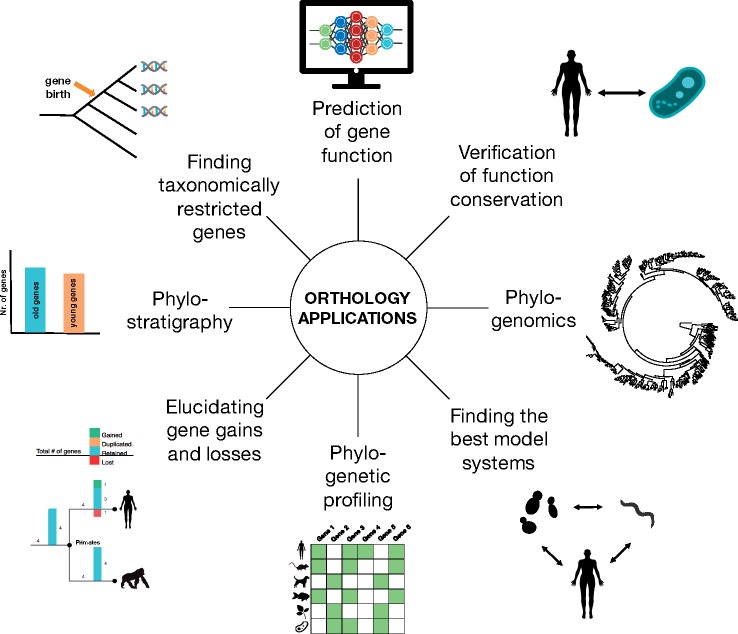
Orthology inference plays a central role in a variety of genomic analyses.

The breadth of uses for orthology predictions continues to increase across the scientific community (fig. 1). Prediction of function, the transfer of knowledge (annotation) from model species to human genes in particular, remains a major application.

The ortholog conjecture—that is, the assumption that orthologs tend to retain their ancestral functions more often than paralogs (Nehrt et al. 2011; Altenhoff et al. 2012)—has the important corollary that function is often conserved between orthologs over long periods of time. Edward Marcotte presented results from experimentally examining the conservation of ortholog function, in vivo, for hundreds of different genes (Kachroo et al. 2015). In his lab, they tested whether orthologous genes can be swapped between two evolutionarily distant species, in this case from human to yeast, which diverged ∼1 Ba. This was successful for 43% of 414 essential yeast genes which were replaceable by 1:1 orthologs in human (lethality when not replaced). Next, they performed a similar analysis from *Escherichia coli* to yeast, which diverged >2 Ba. Due to the larger divergence, the number of essential 1:1 orthologs between the two species was lower, but the proportion of functionally conserved orthologs was even higher (31 out of 51) (Kachroo et al. 2017). These studies show how orthologous genes can retain their function over billions of years of evolution.

Another emergent application of orthology is to find the best model system for a given physiological problem. For example, a species such as ferret (*Mustela putorius furo*) may be a better model organism when studying human respiratory disease than the “go-to” animal mouse, despite having diverged earlier. This is because when looking at the protein sequence divergence for all orthologs between human and mouse versus the divergence of all orthologs between human and ferret, the ferret protein sequences are closer to their human counterparts for 75% of all orthologs (Peng et al. 2014). The assumption that the more conserved the orthologs are, the more the corresponding physiological processes are similar, can be useful to identify a good model organism for that particular physiological pathway.

Orthologs can be used to make phylomes, which are complete collections of phylogenetic trees for each gene encoded in a given genome. These phylomes can be used to detect polyploidization, that is, whole genome duplication events. Phylomes were used by Marcet-Houben and Gabaldón (2015) to detect and analyze polyploidization events through two different approaches: first by calculating the duplication frequency, and second by performing a topology analysis. Through these means they were able to determine that the whole genome duplication that happened in the yeast lineage was actually a hybridization. This phylogenomics approach allows for identifying and disentangling duplication versus hybridization processes, an important application for the polyploidy community.

Although a main goal of orthology prediction is to find genes which have been evolutionarily conserved over long periods of time, the inverse problem of finding taxonomically restricted genes could provide insight into functional evolutionary innovation. Using a phylostratigraphy method combining protein and transcript sequences from 30 different mammalian species, Jose Luis Villanueva-Canas et al. found ∼6,000 mammal-specific gene families (Villanueva-Canas et al. 2017). The taxonomically restricted genes in these families tend to have a high isoelectric point, be short, expressed at a low-level, tissue-specific, enriched in skin and testis, and very few thus far have Gene Ontology (GO) annotations. Those that have GO terms assigned are enriched for terms describing immune response, reproduction, and protein secretion. This potentially sheds light on how and what types of new genes have arisen recently in mammals.

The estimation of how and when genes arise may be dependent on the orthology inference algorithm. In another phylostratigraphy example, gene ages of human proteins were determined using 13 orthology inference algorithms (Liebeskind et al. 2016). Gene age was defined as the age of the last common ancestor in the inferred orthology group. The different orthology inference methods were classified into two groups: 1) methods that found most gene births to be at the vertebrate last common ancestor, and 2) methods that found most gene births to be much older, dating back to the eukaryotic last common ancestor. Although tree-based methods tended to fall in the “old” group and graph-based methods in the “young” group, factors such as systematic error was found to play a large role.

Orthology inference can be used to elucidate where/when (relative to known speciation events) gene duplication and loss events occurred in specific gene families. Several tools are now available to do this for different platforms. For example, pyHam is a python library for handling orthoXML files containing Hierarchical Orthologous Groups (HOGs) (Train et al. 2019). Haiming Tang presented a software tool for parsing gene trees to infer changes in ancestral genome content along specific branches of the species tree, and discussed applications in ancestral genome reconstruction (Huang et al. 2019). HieranoiDB (Kaduk et al. 2017) allows for online browsing of “ortholog trees” to see duplications and speciation events within a gene family. This tracking of gene gains and losses is the basis for another application, phylogenetic profiling, which is a guilt-by-association method for assigning functions based on similar patterns of presence or absence of orthologs between species. For instance, the online tool PhyloProfile can be used for integrating, visualizing, and exploring phylogenetic profiles (Tran et al. 2019). In addition to viewing the presence/absence patterns across many species, PhyloProfile allows for viewing complementary data, like sequence similarity between orthologs, similarities in their domain architecture, or differences in functional annotations.

Prediction of gene function remains one of the major applications of orthology inference. However, prediction at a large scale is only in its early stages, and its accuracy has been challenging to assess (Gillis and Pavlidis 2013). The talks at QFO5/SMBE-EGF showed substantial progress on function prediction. Orthologs have long been postulated to share conserved functions (Tatusov et al. 1997), and, as described earlier, more recently validated in the lab. If function is conserved across orthologs, more so than across other homologs, then the extensive experiments performed in various “model organisms” such as *Caenorhabditis elegans* (nematode worm), fruit fly (*Drosophila melanogaster*), mouse, rat, zebrafish, and the yeasts *Saccharomyces cerevisiae* and *Schizosaccharomyces pombe*, can be used to elucidate, for example, human gene function. Indeed, Marcotte’s more recent experiments have shown that gene function is retained even in some cases where a human ortholog cannot functionally complement a yeast protein: a single amino acid change in a human proteasome subunit is sufficient to restore its function in yeast. Paul Thomas presented ongoing work in his group to develop an algorithm that reproduces function predictions made by expert biocurators when modeling gene function evolution through gene families (Gaudet et al. 2011). Christophe Dessimoz discussed his group’s efforts to use the sequence divergence of orthologous genes between genomes, to identify the best model organism for studying a given biological system. Sofia Forslund described the eggNOG-mapper algorithm (Huerta-Cepas et al. 2017) for inferring function on a genomic scale using pairwise orthology predictions, including extensive validation of the accuracy of these inferences.

Orthology inferences can be integrated with diverse sources of functional data, such as gene expression patterns and mutation phenotypes, to predict different aspects of gene function; QFO5/SMBE-EFG featured talks on two recent integration efforts, from Norbert Perrimon on the Gene2Function system (Hu et al. 2017), and Erik Sonnhammer on the FunCoup (Ogris et al. 2018) functional association network resource. Finally, using orthology to create phylogenetic profiles that show concerted gains and losses of genes in different lineages, Odile Lecompte was able to identify additional genes involved in cilium biogenesis and function (Nevers et al. 2017).

## Methods: the New, the Updated, and the Meta

The applications of orthology depend crucially on the quality of the inferences. There are a plethora of orthology inference methods, and ongoing development includes new methods, updates of existing methods, as well as some meta-methods.

While most ortholog prediction methods operate on the basis of genes as the unit of evolution, domains as units might be more precise. This is due to the independent functionality of the domains, and their ability to create new genes by exon shuffling or domain promiscuity, creating complex homology relationships (Gabaldón and Koonin 2013). Thus, domains could be a more correct functional unit of orthology. This idea has been implemented by Kaduk and Sonnhammer, who have introduced a new tool called Domainoid (https://bitbucket.org/sonnhammergroup/domainoid; Last accessed April 2019). It makes orthology inferences on domains defined by Pfam, to capture orthology between genes that have undergone domain rearrangements and would not be detected with full sequence-length methods. Between *Danio rerio* and *Homo sapiens*, Domainoid’s protein-level ortholog pairs overlap by 60–80% with full-length InParanoid pairs, depending on the fraction of domain-level orthologs required for support. Domainoid should thus be seen as a complement to full-length approaches, in particular useful for detecting discordant domain orthologs, that is, where different domains on the same protein have different evolutionary histories.

A new method was introduced for improving gene phylogenies, called ProfileNJ (Noutahi et al. 2016). It exploits the knowledge from the species tree to correct weakly supported branches on the gene tree, using sequence information in addition to duplication and loss events. More recently, a new method for orthology inference called HyPPO was developed (Lafond et al. 2018), which combines elements of both tree-based and clustering-based approaches: tree-based methods are used to define a set of primary orthologs (also called isoorthologs, Swenson and El-Mabrouk 2012 or least-diverged orthologs, Mi et al. 2010), which are expanded using clustering to include in-paralogs.

With the abundance of different techniques for inferring orthologs, meta-methods, which combine predictions from several methods, are an emerging trend. Published meta-methods can be based on the intersection (e.g., MetaPhOrs, Pryszcz et al. 2011; MARIO, Pereira et al. 2014), union (e.g., HCOP, Eyre et al. 2007; OrthoList, Shaye and Greenwald 2011), or weighted combinations (e.g., DIOPT, Hu et al. 2011; ORCAN, Zielezinski et al. 2017) of predictions from different individual methods. Meta-methods can increase the robustness of ortholog predictions by compensating for deficiencies of each individual method, though there seem to be rapidly diminishing returns as more individual methods are added (Pryszcz et al. 2011; Kim et al. 2018). Machine learning methods have been applied to ortholog meta-analysis: WORMHOLE (Sutphin et al. 2016) makes predictions specifically of least-diverged orthologs using inferences from 14 different individual orthology methods, by training a support-vector machine to generalize data from PANTHER (Mi et al. 2010).

Additionally, well-established methods have been updated, mainly to improve the efficiency of the algorithms in terms of runtime, or to account for distant homologs. For example, the Microbial Genome Database for Comparative Analysis’ (Uchiyama et al. 2019) new pan-genome based analysis procedure starts by selecting representative genes in each species for each orthologous group. Then, it identifies a representative gene within each genus. The representative gene is selected based on several criteria, including not being a partial or split gene, the length being close to median gene length, and that the gene is not an outlier in terms of phylogenetic distance. This method allows for running fewer comparisons, saving computational power. In eggNOG (Huerta-Cepas et al. 2016), the sequence search tools DIAMOND (Buchfink et al. 2015) and HMMER (Eddy 2011) were both implemented to take into account close and distant homologs (or alternatively, well- or poorly sampled clades). OMA was updated as well with a new algorithm to take into account rapidly evolving and duplicated genes (Train et al. 2017). Other improvements come from the new “bottom up” method for constructing HOGs in a faster time.

## QFO as a Resource for the Wider Scientific Community

The QFO Consortium has continued to expand its interactions among members since its inception in 2009. At QFO5/SMBE-EGF, consortium members took an additional step toward serving the wider community of orthology users: annual, benchmarked releases from multiple methods across a consistent set of protein-coding genes.

One achievement of the QFO in recent years was to establish an online benchmarking tool for orthology prediction. This was motivated by needing a way to compare methods, yet not knowing the ground truth for ortholog predictions. Such a service was collaboratively created at http://orthology.benchmarkservice.org; Last accessed April 2019 (Altenhoff et al. 2016), and here anyone can upload ortholog predictions that will be subjected to 20 different benchmarks. The benchmarks are phylogeny-based or function-based, and a user can compare their performance to other methods such as PhylomeDB, InParanoid, OMA, Ensembl Compara, Hieranoid, PANTHER, MetaPhOrs, OrthoInspector, eggNOG, SonicParanoid. Since the publication, 530 jobs have been submitted.

Adrian Altenhoff presented improvements to the service that allow ortholog providers to perform benchmarking on yearly updated data sets. It utilizes a selection of the most up-to-date protein sets available from the UniProt Reference Proteomes (The UniProt Consortium 2017), recently expanded from 66 to 78 proteomes as presented by Alan Sousa da Silva. This was done to broaden the species coverage, by using the Proteome Priority Score (Chen et al. 2011) to identify the best species candidate for a given phylum. Salvador Capella-Gutierrez presented a design for an updated, extensible orthology benchmarking service based on OpenEBench (https://openebench.bsc.es; Last accessed April 2019), supported by the ELIXIR initiative (Capella-Gutierrez et al. 2017).

Building on these services, QFO Consortium members that develop orthology inference methods agreed to update their predictions to cover the same set of protein sequences, and submit them to the benchmark service. In addition, these predictions are now made available from a central location to facilitate the usage of these sets by a wider community of users, such as in meta-analysis across multiple methods, and for comparison between different methods. As of April 2019, the updated, benchmarked ortholog inferences (on the 2018 benchmark) are available for download at the orthology benchmarking service website (https://orthology.benchmarkservice.org; Last accessed April 2019) for nine methods: InParanoid (Sonnhammer and Östlund 2015), Hieranoid 2 (Kaduk and Sonnhammer 2017), OMA Groups (Altenhoff et al. 2018), OMA GETHOGs (Altenhoff et al. 2013), OrthoInspector (Nevers et al. 2019), PANTHER all, PANTHER LDO only (Mi et al. 2019), best bidirectional hit (BBH), and reciprocal smallest distance (RSD). The inferences from all these methods are made on the same set of sequences from the UniProt reference proteomes (UniProt Consortium 2019), which removes the major longstanding barrier to integrating and comparing inferences across different methods: the use of different proteomes with different identifiers by different inference methods.

Although the online benchmarking service is a valuable resource, one limitation is that all the benchmarks are performed on pairwise orthologs as input. For those methods which output orthologous groups, the benchmarking service first reduces them to their pairs to perform the tests. Therefore, new ortholog group-based or labeled gene tree-based benchmarks are needed in the ortholog community. There were several other suggestions for new benchmarks at the QFO5/SMBE-EGF meeting, including: reference families, “large-scale” benchmarks that make use of all data, using a score derived by a meta-method technique, a gene order (synteny) conservation score as a way to identify high-quality orthologs (Patricio et al. 2017), and more function-based and consistency-based tests.

Other possible functional benchmarks are those used during the development of eggNOG-mapper (Huerta-Cepas et al. 2017). For example, benchmarking orthology-based GO predictions against simple homology-based BLAST (Altschul et al. 1990) and InterProScan (Finn et al. 2017) GO predictions was done by determining the number of true positives and true negatives with regards to the best-curated Gene Ontology subsets. Those predictions which were experimentally validated were considered true positives; whereas those with a GO term with taxonomy limitations were considered as true negatives, for example, fin development assigned to a plant gene. The ongoing dialog between functional description ontology developers and orthology inference developers will further improve such approaches in the future.

Advances were also reported on the Orthology Ontology, which enables the use of semantic web tools on orthology inferences. Hirokazu Chiba presented a tour of the Orthology Ontology repository on GitHub (https://github.com/qfo/OrthologyOntology; Last accessed April 2019), including software tools for converting the standard OrthoXML format to RDF, and SPARQL queries to retrieve pairwise orthologs from an RDF triple store (from Jesualdo Tomas Fernandez-Breis).

## Challenges in Orthology Inference

Challenges and limitations to orthology inference remain. As Fitch (2000) noted, some problems are due to differences in terminology, whereas some are due to the complex nature of certain evolutionary scenarios.

Gene conversion can affect orthology inference and interpretation. Bryan Dighera, Arbel Harpak, Xiang Ji reported on nonallelic gene conversion, or the process of copying the sequence of one paralog to replace the sequence of its paralog at another locus. This can cause concerted evolution, where the paralogs look closer in sequence than the related gene family in another species, even though the duplication actually took place earlier than the speciation. This might be a relatively common phenomenon and should be properly tested for. Additionally, repeated duplication can lead to large, diverse gene families. Patricia Babbitt noted that approximately one-third of enzyme superfamilies are functionally diverse, which makes function prediction challenging due to different functions emerging in different subclades. Juan Felipe Ortiz spoke about clusters of tandemly duplicated genes (CTDGs), which are genomic regions with a statistically significant higher number of duplicates than a typical genomic region of the same length. The clear and standard definition will make it easier to implement algorithms for identifying CTDGs.

High rates of sequence divergence can also have an effect on the ability to recognize evolutionary relationships between genes. Ingo Ebersberger discussed his group’s work on calculation of a protein’s evolutionary traceability (Jain et al. 2019), and the effects of traceability on ortholog inference.

The clarity of definitions is an important point when it comes to homology analysis. For the orthology community it is important to establish evolutionarily precise definitions for terms that linguistically have been used more flexibly. For instance, Dannie Durand presented a definition and classification of xenologs as pairs of genes where their history since divergence includes a HGT event. Yan Wang later reported a recently identified xenolog example that an insect gut fungus (*Zancudomyces culisetae*) can encode a mosquito-like polyubiquitin but its original fungal copy was lost (Wang et al. 2016). Natasha Glover presented an evolutionarily precise definition of homoeology: the relationship between genes which diverged by speciation, yet were brought back together in the same species via hybridization followed by whole genome duplication. Homoeologs can thus be thought of as orthologs between subgenomes in an allopolyploid (Glover et al. 2016).

Another type of challenge in orthology inference is the high computational demand of comparing hundreds or thousands of proteomes with each other. This Big Data problem was discussed and various solutions are proposed. Perhaps the most straightforward solution is to replace BLAST, which is used by many graph-based methods, by a much faster homology search tools such as MMseqs2 (Steinegger and Söding 2017) or DIAMOND (Buchfink et al. 2015). Another approach under development is the SIBLINGS resource which shares precomputed all-against-all similarity scores between reference proteomes. Finally, some inference algorithms such as Hieranoid are designed to achieve near linear scaling.

For the QFO Consortium, the next steps will take advantage of the fact that an increasing number of different orthology methods have now been applied to a consistent, comprehensive set of protein-coding genes in 78 taxonomically diverse organisms. Future opportunities include: comparing methods in detail to understand when they agree or differ, increasing the number of organisms in the benchmark, and helping to improve the quality of the UniProt reference proteomes by identifying unexpectedly missing genes or errors in predicted protein sequences that can cause errors in orthology inference.

## Conclusion

Taken together, it was clear from the QFO5/SMBE-EGF meeting that the orthology research community has matured toward an initial “production stage.” Basic axioms of the field have been repeatedly tested, and have become standard practice even across independent teams. Multiple generations of orthology inference tools exist, and previously recognized standards are now being implemented in practice. Algorithms are being developed that focus increasingly on nonstandard evolutionary complexities and previously unasked questions, as much of orthology analysis and applications can now be considered routine. Therefore, more and more effort can be focused on the next phase of challenges and edge cases, both technical and biological, opening new research subfields in the process.
